# Site-specific performance of ^68^Ga-DOTATATE PET/CT in detecting tumors with ectopic adrenocorticotropic hormone secretion

**DOI:** 10.3389/fonc.2023.1204963

**Published:** 2023-06-30

**Authors:** Junhu Wang, Zhonghua Luan, Ting Li, Xiaodong Guan

**Affiliations:** ^1^ Department of Nuclear Medicine, Yuncheng Central Hospital of Shanxi Province, Shanxi, China; ^2^ Department of Pathology, Yuncheng Central Hospital of Shanxi Province, Shanxi, China; ^3^ Department of Urology Surgery, Yuncheng Central Hospital of Shanxi Province, Shanxi, China

**Keywords:** ectopic adrenocorticotropic hormone secretion, 68 Ga-DOTATATE, PET/CT, extrapulmonary, pulmonary

## Abstract

**Objective:**

The aim of this study was to assess the detection ability of ^68^Ga-DOTATATE in pulmonary versus extrapulmonary tumors with ectopic adrenocorticotropic hormone secretion (EAS).

**Methods:**

Images of ^68^Ga-DOTATATE PET/CT from 74 patients with suspected EAS were retrospectively reviewed. EAS tumors were confirmed in 39 patients through surgical resection or biopsy. Image findings were compared with the histopathological results.

**Results:**

EAS tumors were pathologically confirmed *via* surgery or biopsy in 39 patients. Among those 39 patients, 25 were with pulmonary neuroendocrine tumors (NETs), and the remaining 14 were with extrapulmonary NETs. ^68^Ga-DOTATATE PET/CT correctly identified the tumor in 26 patients, rendering an overall detection rate of 66.7%. On a site-based analysis, ^68^Ga-DOTATATE PET/CT correctly identified the EAS tumor in 13 of 25 patients with pulmonary NETs, yielding a detection rate of 52%; for the 14 patients with extrapulmonary NETs, ^68^Ga-DOTATATE PET/CT correctly identified the EAS tumor in 13, yielding a detection rate of 92.9%. The detection rate of ^68^Ga-DOTATATE was significantly higher in extrapulmonary NETs than in pulmonary NETs (92.9%% *vs*. 52%, P = 0.013). For the 13 patients with positive pulmonary NETs, the tumor SUVmax ranged from 1.1 to 7.4 with an average SUVmax of 3.1 ± 2.1. For the 13 patients with positive extrapulmonary NETs, the tumor SUVmax ranged from 2.7 to 21.8 with an average SUVmax of 9.9 ± 6.3. The tumor SUVmax was significantly higher in extrapulmonary tumors than pulmonary tumors (P = 0.015). The tumor size was smaller in pulmonary tumors than in extrapulmonary tumors, while the difference was not significant (P = 0.516).

**Conclusion:**

^68^Ga-DOTATATE showed site-specific difference in detecting tumors with EAS secretion. Specifically, ^68^Ga-DOTATATE performed better in the extrapulmonary EAS tumors than in pulmonary ones with both higher detection rate and uptake. Combination of anatomic imaging techniques are necessary for the correct diagnosis of pulmonary EAS tumors.

## Introduction

Cushing’s syndrome is a rare disease. It is classified into adrenocorticotropic hormone (ACTH)-dependent Cushing’s syndrome, which accounts for 80% of cases, and ACTH-independent Cushing’s syndrome, which accounts for 20% of cases (1, 2). Ectopic adrenocorticotropic hormone secretion (EAS) is a rare cause of ACTH-dependent Cushing’s syndrome (3). The tumors responsible for EAS can originate from various sites of the body, and the most commonly responsible tumors are pulmonary neuroendocrine tumor (NET), followed by NET in other extrapulmonary locations (thymus, pancreas, rectum, appendix), and pheochromocytomas in rare cases (4). Correct identification of the EAS source is crucial as surgical resection of the EAS tumor can be curative for hypercortisolism, and may avoid unnecessary adrenalectomy and lead to a favorable prognosis (1, 5).

Conventional anatomic imaging, including CT and MR, is usually recommended as the initial imaging modality for identifying the EAS tumor (3, 6). Functional imaging, Gallium-68 labeled somatostatin receptor PET/CT (^68^Ga-SSTR PET/CT) has been proposed as a highly sensitive imaging modality for NET in general, and has been shown to be superior to other imaging methods in identifying the EAS tumor (3, 7, 8). As mentioned before, NET responsible for EAS can originate from various locations. Previous studies on the performance of ^68^Ga-SSTR PET/CT generally included NETs of various origins. Marked disease heterogeneity exists in NET; most gastroenteropancreatic (GEP) NETs have high SSTR expression while only a proportion of pulmonary NET express SSTR at sufficient levels (9, 10).

Based on practical experience and literature report, we speculated that ^68^Ga-DOTATATE might perform better in extrapulmonary NETs than pulmonary ones responsible for EAS. In order to confirm this speculation, in the present study, we retrospectively reviewed the EAS patients evaluated by ^68^Ga-DOTATATE to determine whether ^68^Ga-DOTATATE performed differently between pulmonary and extrapulmonary NETs responsible for EAS.

## Material and methods

### Patients

We retrospectively reviewed the medical records from 74 patients who underwent ^68^Ga-DOTATATE PET/CT between July 2014 and December 2022 for localizing the source of EAS in our hospital. Of the 74 patients, the tumor responsible for EAS was histopathologically proven in 39 patients through surgery or biopsy. For these 39 patients, ^68^Ga-DOTATATE PET/CT was performed 2 weeks to 3 months before surgery or biopsy. The ^68^Ga-DOTATATE PET/CT images of these 39 patients were further analysed and compared with the results of the pathological examinations. The histological diagnosis of gastroentero-pancreatic NET was made according to the 2019 WHO classification, and the histological diagnosis of thoracic NETs (lung, thymus, heart) was made according to the 2021 WHO classification. The chart review and image analysis for the patients in this study were approved by the institutional review board of our hospital (Approval number: YXLL20230021; date: 15 Feb 2023), patient consents were not required due to the retrospective design.

### ^68^Ga-DOTATATE PET/CT imaging

Before ^68^Ga-DOTATATE PET/CT, the use of long-acting somatostatin analogue was not allowed. PET/CT acquisitions were performed with dedicated PET/CT scanners (Biograph 64 Truepoint True V, Siemens; Discovery 690, GE Healthcare). PET/CT scans were acquired from the head to the mid-thigh in a 3D mode (2 minutes per bed position). The scans were started 40˜60 minutes after the intravenous administration of ^68^Ga-DOTATATE at a dose of 74˜148 MBq (2˜4mCi).

### Image interpretation and statistical analysis

The ^68^Ga-DOTATATE PET/CT images were interpreted by 2 experienced nuclear medicine physicians. The images were visually and semi-quantitatively analysed. Lesions with tracer uptake higher than the surrounding tissue were considered positive, and lesions with tracer uptake similar to or less than the surrounding tissue were considered negative. The maximum standard uptake value (SUV_max_) and size (longest diameter) of the tumors were recorded.

The statistical analyses were performed using SPSS Statistics (version 21.0, IBM SPSS Inc., IBM, Chicago, IL, USA). Continuous data are expressed as mean ± standard deviation. The detection rate of ^68^Ga-DOTATATE PET/CT between pulmonary and extrapulmonary NETs was compared using χ2 test. The difference of tumor SUVmax and size between pulmonary and extrapulmonary NETs was compared using nonparametric analysis. A *P* value less than 0.05 was considered statistically significant.

## Results

Among the 39 patients with pathologically confirmed EAS tumors, 25 were with pulmonary NETs, and the remaining 14 were with extrapulmonary NETs. For the 25 patients with pulmonary NETs, 15 were diagnosed with typical carcinoids (TC), the remaining 10 were with atypical carcinoids (AC). The Ki-67 proliferation index of patients with pulmonary NETs ranged from 1% to 10% with a median value of 2%. The mitotic index data was available for 6 patients, and they were 1, <2, <2, <2, 3, and 4 mitoses/2 mm^2^, respectively. For the 14 patients with extrapulmonary NETs, 3 were diagnosed with a thymic NET, 4 with a mediastinal NET, 2 with a pancreatic NET, 1 with a rectal NET, 1 with an intramyocardial NET, 1 with an appendiceal NET, and 2 with pheochromocytoma. The Ki-67 proliferation index ranged from 1% to 70% with a median value of 5%. The mitotic index data was available for 2 of the patients, and they were <2 mitoses/2 mm^2^ and 4 mitoses/2 mm^2^, respectively. Immunohistochemistry results for ACTH were positive in all patients. The clinical characteristics of patients are listed in **Table 1**.

**Table 1:** Clinical characteristics of patients with confirmed ectopic adrenocorticotropic hormone secretion.

	Pulmonary NETS	Extrapulmonary NETs	P value
**Number of patients**	25	14	–
**Age (years)**	37.8 ± 16.3	42.9 ± 16.7	0.309
**Sex (F/M)**	16/9	7/7	–
**Ki-67 proliferation index (median, range)**	3.5 ± 2.5%, (1% ˜ 10%)	12.7 ± 20.9, (1% ˜ 70%)	0.394
**Tumor size (cm)**	1.1 ± 0.6	1.3 ± 0.5	0.516
**Tumor SUVmax**	3.1 ± 2.1	9.9 ± 6.3	0.015
**Detection rate of ^68^Ga-DOTATATE**	52%	92.9%	0.013

**Figure 1:**
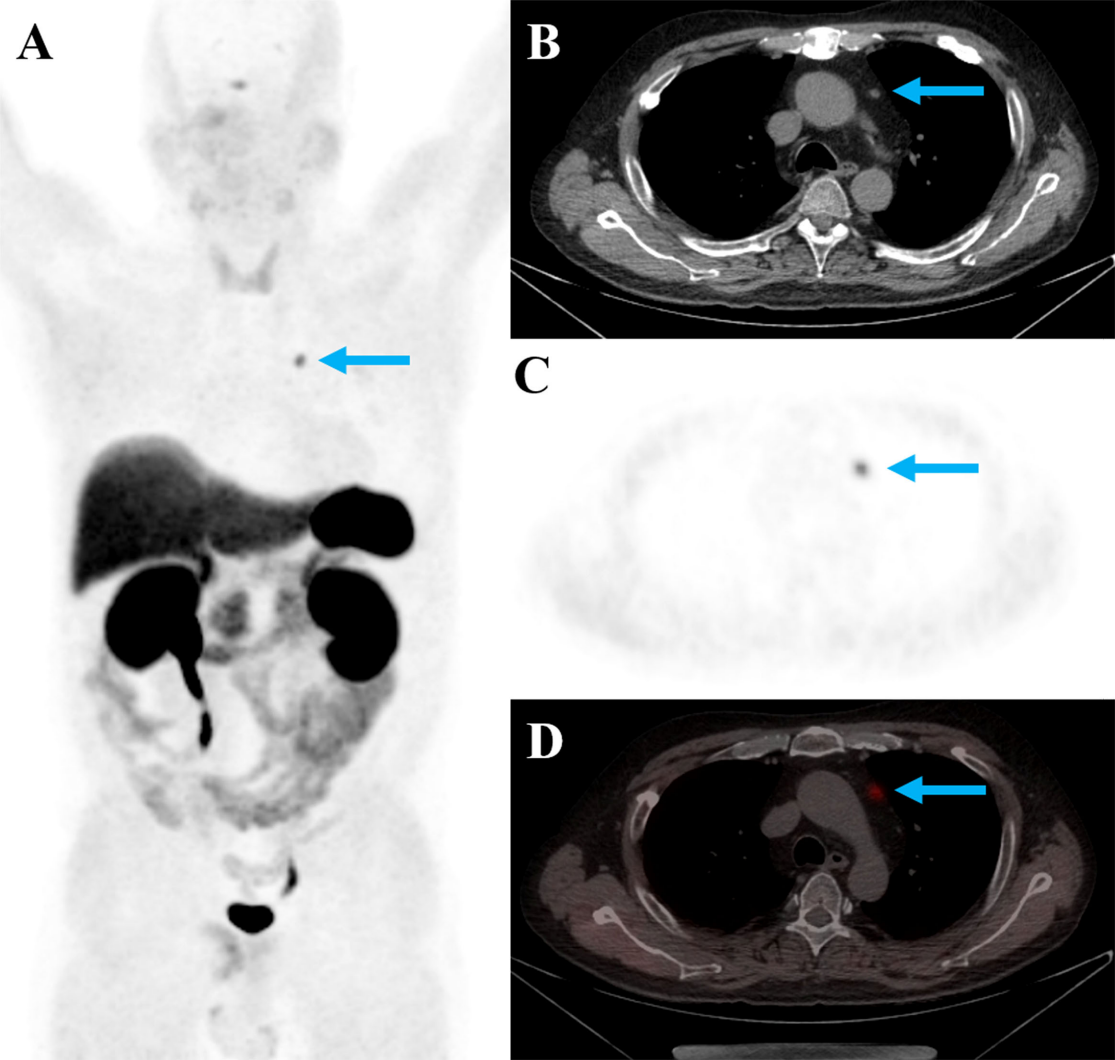
A 53-year-old man with EAS showed positive ^68^Ga-DOTATATE uptake in the thymic nodule (**A–D**, blue arrows; tumor SUVmax: 5.2; tumor size: 0.5cm). The nodule was surgically resected, and was pathologically confirmed to be thymic neuroendocrine tumor (G1, Ki-67 index, 1%).

^68^Ga-DOTATATE PET/CT correctly identified the EAS tumor in 26 patients, rendering an overall detection rate of 66.7%. On a site-based analysis, we found that for the 25 patients with pulmonary NETs, ^68^Ga-DOTATATE PET/CT identified the tumor in 13 patients, yielding a detection rate of 52%. While for the 14 patients with extrapulmonary NETs, ^68^Ga-DOTATATE PET/CT identified the tumor in 13, yielding a detection rate of 92.8%, which was significantly higher than that for pulmonary NETs (P = 0.013). The only patient with extrapulmonary NET undetected by ^68^Ga-DOTATATE PET/CT was with thymic carcinoid (tumor size, 0.8cm; **Figure 1**). The detection rate of ^68^Ga-DOTATATE in pulmonary TC and AC was 46.7% (7/15) and 60% (6/10), respectively, and there was no statistically significant difference (P = 0.688).

For the 13 patients with positive pulmonary NETs, the tumor SUVmax ranged from 1.1 to 7.4 with an average SUVmax of 3.1 ± 2.1. For the 13 patients with positive extrapulmonary NETs, the tumor SUVmax ranged from 2.7 to 21.8 with an average SUVmax of 9.9 ± 6.3. The average SUVmax was significantly higher in extrapulmonary tumors than pulmonary tumors (P = 0.015). We also compared the tumor size of all patients with pulmonary and extrapulmonary NETs. The tumor size of pulmonary NETs ranged from 0.5 to 3.0 cm with an average size of 1.1 ± 0.6 cm (median, 1.0 cm). The tumor size of extrapulmonary NETs ranged from 0.5 to 2.5 cm with an average size of 1.3 ± 0.5 cm (median, 1.4 cm). The comparison revealed that the tumor size of extrapulmonary NETs was larger than pulmonary NETs (1.1 ± 0.6 cm *vs*. 1.3 ± 0.5 cm, P = 0.516).

## Discussion

The identification of tumors responsible for EAS has always been a challenge. Previous studies have demonstrated that ^68^Ga-DOTATATE PET/CT is an effective imaging modality in localizing EAS tumors (11, 12). To date, our study includes the largest number of patients with confirmed EAS tumors evaluated with ^68^Ga-DOTATATE PET/CT from a single center. Our results revealed an overall detection rate of 66.7% of ^68^Ga-DOTATATE PET/CT for detecting EAS tumor, which is lower than that reported by previous published studies. A recent prospective study by Liu et al. reported a detection rate of 75% of ^68^Ga-DOTATATE PET/CT in 20 patients with histologically confirmed EAS tumors (11). The reason why ^68^Ga-DOTATATE showed a lower detection rate in our study might be due to the fact that, the proportion of pulmonary NETs (64.1%, 25/39) in our study is higher than theirs (50%, 10/20). This will be further elaborated below. Two recent systematic reviews also reported higher detection rate of ^68^Ga-DOTATATE of 76.1% (n=69) and 81.8% (n=23), respectively (3, 13). However, these two systematic reviews are subject to publication bias. Majority of the articles included were case reports and case series, and cases of false negative ^68^Ga-DOTATATE scans are likely underreported.

The main finding of the present study is that the detection rate of ^68^Ga-DOTATATE PET/CT in extrapulmonary NETs is significantly higher than that in pulmonary ones. This phenomenon is not well known to clinical physicians at present. Still, a similar finding has been mentioned briefly in a previous study by Goroshi et al. They retrospectively reviewed 12 EAS patients evaluated with ^68^Ga-DOTANOC PET/CT, and reported a tendency for ^68^Ga-DOTANOC to have a lower sensitivity for lung lesions (60%, 6/10) than GEP-NETs (100%, 3/3) (12). The study of Liu et al. included 20 patients with pathologically confirmed EAS tumors, which consisted of 10 with pulmonary NETs and 10 with extrapulmonary NETs. Recalculation of their data also revealed a lower detection rate of ^68^Ga-DOTATATE for pulmonary NETs than extrapulmonary ones (60% *vs*. 90%) (11). We also found that the tumor SUVmax of extrapulmonary NETs was significantly higher than that of pulmonary NETs. In Addition, the size of pulmonary tumors tended to be smaller, although not statistically significant, than extrapulmonary tumors. Pulmonary tumors with small size are often subject to partial-volume effect, making it difficult to measure the actual metabolic status (14). It is noteworthy that all pulmonary EAS tumors in this study were detected by chest CT. The finding of ^68^Ga-DOTATATE demonstrating higher detection rate in extrapulmonary NETs than in pulmonary ones is of clinical significance and helps the selection of diagnostic modality. For pulmonary EAS tumor, combination of anatomic imaging techniques is necessary to achieve a correct diagnosis.

By comparing with previously published studies, we also found that, the detection rate of ^68^Ga-DOTATATE for pulmonary NETs (52%) responsible for EAS was lower compared to that reported for nonfunctional ones. A meta-analysis evaluated the diagnostic accuracy of ^68^Ga-SSTR PET/CT in pulmonary carcinoids, and reported that the pooled sensitivity of ^68^Ga-SSTR PET/CT in detecting pulmonary carcinoid was 90%; the range of sensitivity of ^68^Ga-SSTR PET/CT was 79%˜100% (15–19). One reason might be that the pulmonary NETs responsible for EAS were much smaller in size. The average tumor size of pulmonary NETs in the present study was 1.1 ± 0.6 cm. While the average tumor size of nonfunctional pulmonary carcinoids reported in previous studies ranged from 2.7 ± 1.3cm to 4.1 ± 1.9cm (20–22). Another reason might be that hypercortisolism associated with EAS may cause the downregulation of SSTR expression (8, 23, 24). The Ki-67 index was correlated WHO grade (G1, <3%; G2, 3-20%; G3, >20%), and the Ki-67 index mean value of patients depends on the percentage of patients with G1/2/3. The Ki-67 index value of pulmonary NETs was lower than extrapulmonary NETs with no statistically significant difference. This was expected as the pulmonary NETs in our series included 15 typical carcinoids (Ki-67, <3%) and 10 atypical carcinoids (Ki-67, 3-20%), and extrapulmonary NETs included one G3 patient with Ki-67 of 70%.

This study had several limitations. First, the present study was retrospective in design, which might be subject to inherent selection biases. Second, the number of extrapulmonary NETs was relatively small compared to that of pulmonary NETs. Besides, extrapulmonary NETs include tumors of various origins. Thus, we were not able to further investigate the site-specific performance of ^68^Ga-DOTATATE in extrapulmonary NETs. Thirdly, in reviewing of existing literature, we did not find robust evidence to support that pulmonary NETs with EAS have lower SSTR expression than pulmonary NETs without EAS. It is only a speculation that hypercortisolism associated with EAS might cause downregulation of SSTR expression of pulmonary NETs. In future, studies comparing SSTR status in pulmonary NETs with EAS and those without EAS by immunohistochemistry should be carried out. Lastly, our manuscript might contain deficiencies regarding pathological diagnosis, as pathological data of most patients were obtained through retrospective review of the pathological reports. Still, the diagnoses of tumor to be EAS source causing Cushing syndrome were correct as immunohistochemistry results for ACTH were positive in all patients and patients achieved symptoms relief after surgical removal of the tumor.

## Conclusions

^68^Ga-DOTATATE showed site-specific difference in detecting tumors with EAS secretion. Specifically, ^68^Ga-DOTATATE performed better in the extrapulmonary EAS tumors than in pulmonary ones with both higher detection rate and uptake. Combination of anatomic imaging techniques are necessary for the correct diagnosis of pulmonary EAS tumors.

## Data availability statement

The original contributions presented in the study are included in the article/supplementary material. Further inquiries can be directed to the corresponding authors.

## Ethics statement

The studies involving human participants were reviewed and approved by the institutional review board of Yuncheng Central Hospital of Shanxi Province. Written informed consent for participation was not required for this study in accordance with the national legislation and the institutional requirements. Written informed consent was not obtained from the individual(s) for the publication of any potentially identifiable images or data included in this article.

## Author contributions

JW, TL, ZL, and XG contributed to the design and implementation of the research, to the analysis of the results, and to the writing of the manuscript. All authors contributed to the article and approved the submitted version.
